# The mitochondrial‐endoplasmic reticulum co‐transfer in dental pulp stromal cell promotes pulp injury repair

**DOI:** 10.1111/cpr.13530

**Published:** 2023-07-26

**Authors:** Xiaoyi Zhang, Chunmeng Wang, Zihao Zhou, Qi Zhang

**Affiliations:** ^1^ Department of Endodontics Stomatological Hospital and Dental School of Tongji University, Shanghai Engineering Research Center of Tooth Restoration and Regeneration Shanghai China

## Abstract

Dental pulp injury remains a clinical challenge with limited therapeutic approaches. In the present study, we sought to prove that dental pulp stromal cells (DPSCs) mitochondrial transfer could promote dental pulp injury repair and endoplasmic reticulum (ER)‐mitochondrial contacts have a significant regulatory effect on mitochondrial transfer. Healthy DPSCs were co‐cultured directly or indirectly with injured DPSCs in the first molar of 1–2 month SD rats or in vitro. Mitochondrial transfer was observed after 24 h of co‐culture using fluorescence microscopy and live cell workstation. After co‐culture for 1W, 8‐OhdG immunofluorescence, mitochondrial membrane potential and total oxidant status/total antioxidant status were used to detect the mitochondrial function of injured DPSCs before and after mitochondrial transfer. Subsequently, mitochondria‐ER co‐transfer was regulated by modulating mitochondria‐ER binding in healthy DPSCs, and the results of GRP78 and CHOP in DPSCs, and PDI immunofluorescence and haematoxylin and eosin staining of pulp tissue were analysed to clarify the effects of modulating mitochondria‐ER co‐transfer on endoplasmic reticulum stress (ERS), and on pulp injury repair. Fluorescence microscopy and live cell workstation results showed significant mitochondrial transfer between DPSCs. Meanwhile, mitochondrial transfer significantly restored mitochondrial function in injured DPSCs. By modulating mitochondrial‐ER binding, the efficiency of mitochondrial transfer between DPSCs was significantly affected and had an impact on ERS in injured cells. Mitochondrial transfer of DPSCs significantly promotes pulpal injury repair and functional recovery of damaged DPSCs, and mitochondrial transfer of DPSCs is regulated by mitochondria‐ER binding.

## Graphical abstract

Mitofusin2 (Mfn2) regulates mitochondrial‐endoplasmic reticulum (ER) cotransfer between dental pulp stromal cells (DPSCs).

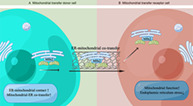

## 1 INTRODUCTION

Pulpitis is one of the most common inflammatory dental diseases, accounting for approximately 24%–44% of emergency dentistry patients and is the leading cause of dental pulp injury and necrosis.^1^, ^2^, ^3^, ^4^, ^5^ As a particular connective tissue, dental pulp has its unique way of repairing injuries, mainly through proliferation and differentiation of dental pulp stromal cells (DPSCs). These cells eventually secrete reparative dentin to repair the pulp‐dentin complex and maintain pulp activity.^6^, ^7^, ^8^ Oxidative stress (OS) impairment of mitochondrial function in DPSCs is considered the primary mechanism affecting pulpal injury repair. Mitochondria are the core organelles of cellular functions that participate in ATP production, cell proliferation, and differentiation.^9^, ^10^ The pulp is surrounded by non‐concessional hard tissue and lacks blood flow, leaving the pulp tissue in a localized hypoxic environment. In injury, the hypoxic environment further induces the accumulation of mitochondrial reactive oxygen species (mtROS) and OS, and the decreasing the activity of antioxidant enzymes such as superoxide dismutase (SOD) and catalase (CAT), ultimately leading to mitochondrial dysfunction.^11^, ^12^, ^13^, ^14^ Therefore, restoring the mitochondrial function of DPSCs early in the injury to avoid mtROS accumulation and OS is the key to successful pulp restoration.^15^

Stem or stromal cells can spontaneously establish targeted mitochondrial transfer channels with injured cells and deliver mitochondria, restoring cellular mitochondrial function and promoting tissue repair.^16^, ^17^ For example, mitochondrial transfer between bone marrow‐derived mesenchymal stromal cells (BMMSCs) can play a significant role in reducing ROS production, increasing energy synthesis, restoring mitochondrial membrane potential and promoting bone repair.^18^ DPSCs are homologous to BMMSCs and have similar multidirectional differentiation abilities and mitochondrial functions, possibly making DPSCs ideal mitochondrial transfer cells.^19^, ^20^ Based on this, we formulate the hypothesis that healthy DPSCs could promote functional recovery of injured DPSCs and pulp repair through mitochondrial transfer.

The process of mitochondrial transfer mainly involves the movement of mitochondria from donor cells to recipient cells through various transfer pathways, including tunnelling nanotubes (TNTs).^21^ TNTs are considered to be the main pathway of mitochondrial transfer between stromal cells, but the mechanism of mitochondrial movement on microtubule structures in TNTs is still not well understood. Previously, it has been observed that mitochondria do not exist freely but form contact with endoplasmic reticulum (ER). Mitochondria‐ER contact not only coordinate signal communication, dynamics, and function between organelles but also regulate protein folding modifications through endoplasmic reticulum stress (ERS) in tissue injury.^22^, ^23^ Meanwhile, researchers have found in osteoblasts that ER and mitochondria linker protein Mitofusin 2 (Mfn2) may be involved in regulating mitochondrial transfer.^24^ This prospective study was designed to investigate whether mitochondria‐ER contact regulates the detachment of mitochondria from the mitochondrial network, allowing them to unbind and enhance their motility, resulting in synchronized movement in DPSCs. Consequently, We propose the hypothesis that by controlling mitochondria‐ER contacts, the primary factors regulating mitochondrial motility and transfer can be identified, ultimately promoting the repair of injured cells through mitochondrial transfer.

## 2 MATERIALS AND METHODS

### 2.1 Pulp injury model establishment

To simulate molar pulp injury, we used 1–2‐month‐old male Sprague–Dawley (SD) rats as experimental subjects to simulate molar pulp injury.^24^ Rats were anaesthetised with 2% sodium pentobarbital (Sigma‐Aldrich). A hemispherical cavity was created in the mesial half of the bilateral maxillary first molars. When red pulp was visible at the bottom, a sterile dental explorer and K‐files #40 were used to expose the entrance to the medullary horn until it reached a width of 1 mm. To ensure haemostasis, a cotton ball was pressed into the cavity. The pulp‐exposed cavity was then covered with gelatin sponges (JinLing Pharma) soaked in different concentrations of LPS (0, 5, and 10 mg/mL, Cayman Chemical) for 30 min. Afterward, we restored all cavities with 1 mm of BP plus (Innovative Bioceramix) and composite resin (Kerr Corp). OS and mitochondrial function‐related indices were observed 1 week after implantation, and reparative dentin formation was observed after 4 weeks.^25^ The protocol for the animal experiment was approved by the Ethical Committee ([2021]‐DW‐21). The exposure sites were evaluated using an optical microscope with 10×, 20×, 40× and 100×‐objective lenses (Leica Microsystems ICC50 HD; Nussloch) and an image capture system (Leica, version 2.10, 2012). Three rats per group, with no exclusion.

### 2.2 In vivo mitochondrial transfer model establishment

In the mitochondrial transfer model, after adding LPS to the pulp exposure site, healthy rat dental pulp stromal cells (rDPSC) were treated (1 × 10^6^) on the pulp‐exposed cavity in 20 μL of PBS as donor cells for in vivo mitochondrial transfer. The rDPSCs were labelled with mito‐mRFP (Beyotime) and CFSE‐GFP (Beyotime) prior to implantation to distinguish them from in vivo receptor rDPSCs. Finally, the cavities were closed with BP plus and composite resin. No scaffold material was used for the implantation of rDPSCs to limit the long‐term survival of the implanted cells and to ensure that their mechanism of action is paracrine. On days 1, 7 and 28 after the operation, animals were executed by over‐anaesthesia with sodium pentobarbital (120 mg/kg body weight) and intracardiac perfused with 4% paraformaldehyde (PFA, pH 7.2–7.4). Mitochondrial transfer was observed 1 day after implantation of rDPSCs. Donor cell mito‐mRFP enters the recipient cell, indicating that mitochondrial transfer occurs. The protocol for the animal experiment was approved by the Ethical Committee ([2021]‐DW‐21). Three rats per group, with no exclusion.

### 2.3 Sample collection and histological analysis

After immersion in 4% PFA for 24 h, at 4°C, the molars were immersed in 10% EDTA solution for demineralization. The EDTA solution was changed every 3 days to ensure the demineralization effect and antigenic activity. We dehydrated the samples using an ethanol concentration gradient and embedded them in paraffin. Following de‐paraffinization and re‐hydration, the samples were sectioned at a thickness of 2 μm, stained with haematoxylin–eosin (HE, Sangon Biotech), and examined under a microscope (Nikon Eclipse 80i). This method was performed as previously described.^26^

### 2.4 Immunohistochemical staining

Sections were treated with hyaluronidase and goat serum (Beyotime) before immunohistochemical staining. Then the sections were incubated with primary antibody anti‐IL‐1 (1:200; Abcam) at 4°C for 12 h, respectively. After incubation with biotinylated secondary antibodies, sections were detected with a DAB detection kit (Beyotime).

### 2.5 Immunofluorescence staining

Antigen retrieval and goat serum (Beyotime) was applied to the sections for immunofluorescence staining. The sections were then incubated for 12 h at 4°C with anti‐8‐OHdG (1:200, Abcam). After rinsing in PBS and incubating for 1 h with secondary antibodies (1:1000; Invitrogen). The sections were counterstained with 4′,6‐diamidino‐2‐phenylindole (DAPI, Beyotime) and observed under a fluorescence microscope (Nikon Eclipse 80i).

### 2.6 Primary human (h) DPSCs and rat (r) DPSCs isolation and culture

Informed consent was obtained from healthy patients (13–20 years old) from Tongji Stomatology Hospital to provide pulp tissue from healthy premolars for orthodontic therapy or impacted third mandibular molars. To extract single‐cell suspensions, pulp tissue was split into 1 mm^2^ pieces and added with trypsin for 15–45 min. Then we used the essential medium alpha modification (α‐MEM; Hyclone) containing 10% foetal bovine serum (FBS; Hyclone), 100 mg/mL streptomycin and 100 U/mL penicillin to culture the cells. The cell culture environment was strictly limited to 37°C in 5% CO^2^. Rat dental pulp tissue was extracted from 6‐week‐old male SD rat bilateral molars. The subsequent culture process was similar to that of hDPSCs. The technique was used as previously reported.^27^

### 2.7 Cell treatment

DPSCs stimulated with LPS were utilized to simulate the cell injury during the pulpitis pathological process. In detail, DPSCs were primed with 1, 5, and 10 μg/mL LPS (LPS25, Sigma Aldrich) for 24 h, To inhibit mitochondrial transfer and TNT formation, cells were pre‐treated with 1 μM Lat‐B (HY‐16928, MedChemExpress) for 6 h to interfere polymerization and interaction of actin.

### 2.8 In vitro mitochondrial transfer model establishment

To observe the phenomenon of mitochondrial transfer in vitro, we used mito‐mRFP to visualize the mitochondria of healthy DPSCs and CFSE‐GFP to label the cytoplasm of injured DPSCs. Afterwards, the two groups of DPSCs were co‐cultured directly or indirectly via the transwell system for 24 h. The transwell pore size is 1 μm to block the cells and facilitate the passage of mitochondria. Donor cell mito‐mRFP enters the recipient cell, indicating that mitochondrial transfer occurs. DAPI (Beyotime) stains all cell nuclei fixed with 4% paraformaldehyde. The method was performed as previously described.^28^ All images were assembled and analysed using Fiji (National Institutes of Health), and all experiments were performed in passages 2–5.

### 2.9 Mitochondrial transfer analysis

CFSE‐GFP‐labelled damaged DPSCs were co‐cultured in vitro with mito‐RFP‐transfected health under the indicated conditions. In vivo CFSE‐GFP and mito‐RFP co‐transfected cells were co‐cultured with damaged tissues under the indicated conditions. The number of injured cells receiving mitochondrial transfer was subsequently quantified by BD FACSAria™ III (Becton Dickinson).

### 2.10 Flow cytometry analysis of cell proliferation

The DPSCs proliferation cycle was analysed after 24 h of co‐culture. The digested single‐cell suspensions were fixed in pre‐cooled 70% ethanol for 20 min. The samples were then incubated with propidium iodide (PI)/RNase staining solution (Beyotime) for 15 min at room temperature. Finally, cell proliferation was analysed by BD FACSAria™ III (Becton Dickinson).

### 2.11 Alizarin red S and alkaline phosphatase staining

Cells were inoculated into culture plates and cultured in an odontoblastic differentiation medium. The cell odontoblastic differentiation medium consisted of 5 mmol/L β‐glycerophosphate (Sigma‐Aldrich), 100 nmol/L dexamethasone (Sigma‐Aldrich) and 50 μmol/L ascorbic acid diphosphate (Sigma‐Aldrich). The culture medium was changed every 2 days for 3 weeks. Next, we stained the cells with ALP and Alizarin Red S. After fixing the cells in 4% PFA for 20 min, we added ALP solution (Beyotime) or 0.2% alizarin red (Sigma‐Aldrich) to each well and incubated for 10–20 min at room temperature. The method was performed as previously described.^25^

### 2.12 Western blot

First, we extracted total proteins from cells using RIPA buffer (Beyotime) supplemented with protease inhibitor (Thermo Scientific) and determined protein concentration (BCA assay, Thermo Scientific). Proteins were then separated on 10% SDS‐PAGE at 120 V and transferred to PVDF membranes at a current of 300 mA. After being blocked with 5% BSA for 2 h. The PVDF membranes were incubated overnight with the following primary antibodies: anti‐CHOP (1:1000, ab11419, Abcam), anti‐GRP78 (1:1000. ab21685, Abcam), and anti‐β‐actin (1:1000, ab8227, Abcam). The next day, we incubated PVDF membranes with fluorescent secondary antibodies (IRDye‐conjugated goat anti‐rabbit IgG, LI‐COR Biosciences; 1:8000) for 45 min at room temperature and detected by enhanced chemiluminescence (Bright ECL kit, Advansta).

### 2.13 Lentivirus construction and transfection

Mfn2 lentiviral knock‐out, lentiviral overexpression plasmids, and control shRNA were purchased from Sigma (Sigma Aldrich) and used per the recommended conditions with an multiplicity of infection (MOI) of 10.

### 2.14 Intracellular ATP measurement

The ATP assay was performed with an ATP Assay Kit (Beyotime). We added 20 μL of the sample to each well of the 96‐well plate, then mixed rapidly and measured the RLU with a luminometer at least 2 s later.

### 2.15 Measurement of mitochondria membrane potential and ROS evaluation

We used the JC‐1 kit (Beyotime) to assess mitochondrial membrane potential. Injured DPSCs were incubated with JC‐1 for 20 min. Then we performed the ROS Assay Kit (Beyotime) to evaluate mitochondria ROS. Injured DPSCs were set with diluted DCFH‐DA and incubated for 20 min at 37°C in a cell culture incubator.

### 2.16 Total oxidant status (TOS)/total antioxidant status (TAS) analysis

We used the TOS and TAS assay kit to assay DPSCs and dental pulp tissues after 24 h of mitochondrial transfer. We recorded TOS/TAS values to reflect redox status and mitochondrial function.

### 2.17 Analysis of organelle transfer in living cell workstation

Pre‐fluorescently labelled DPSCs and injured DPSCs were inoculated in 24‐well culture plates dedicated for confocal microscopy and placed on a live cell workstation observation table with an observation interval of 15 s to capture the mitochondrial movement and transfer process. All images were assembled and analysed using Fiji (National Institutes of Health).

### 2.18 Antioxidant enzyme activity assays

The antioxidant capacity of the DPSCs was evaluated by assaying activities of SOD and CAT using the Superoxide Dismutase Activity Assay kit (Abcam) and Catalase Assay Kit (Abcam) according to manufacturer's instructions.

### 2.19 Statistical analyses

Data were analysed using standard t‐test and ANOVA. *p* < 0.05 was considered significant. Numerical estimates and analysis for graphing were obtained using Graphpad Prism version 7 (Graphpad Inc.).

## 3 RESULTS

### 3.1 Mitochondrial transfer promotes pulpal injury repair

**FIGURE 1:**
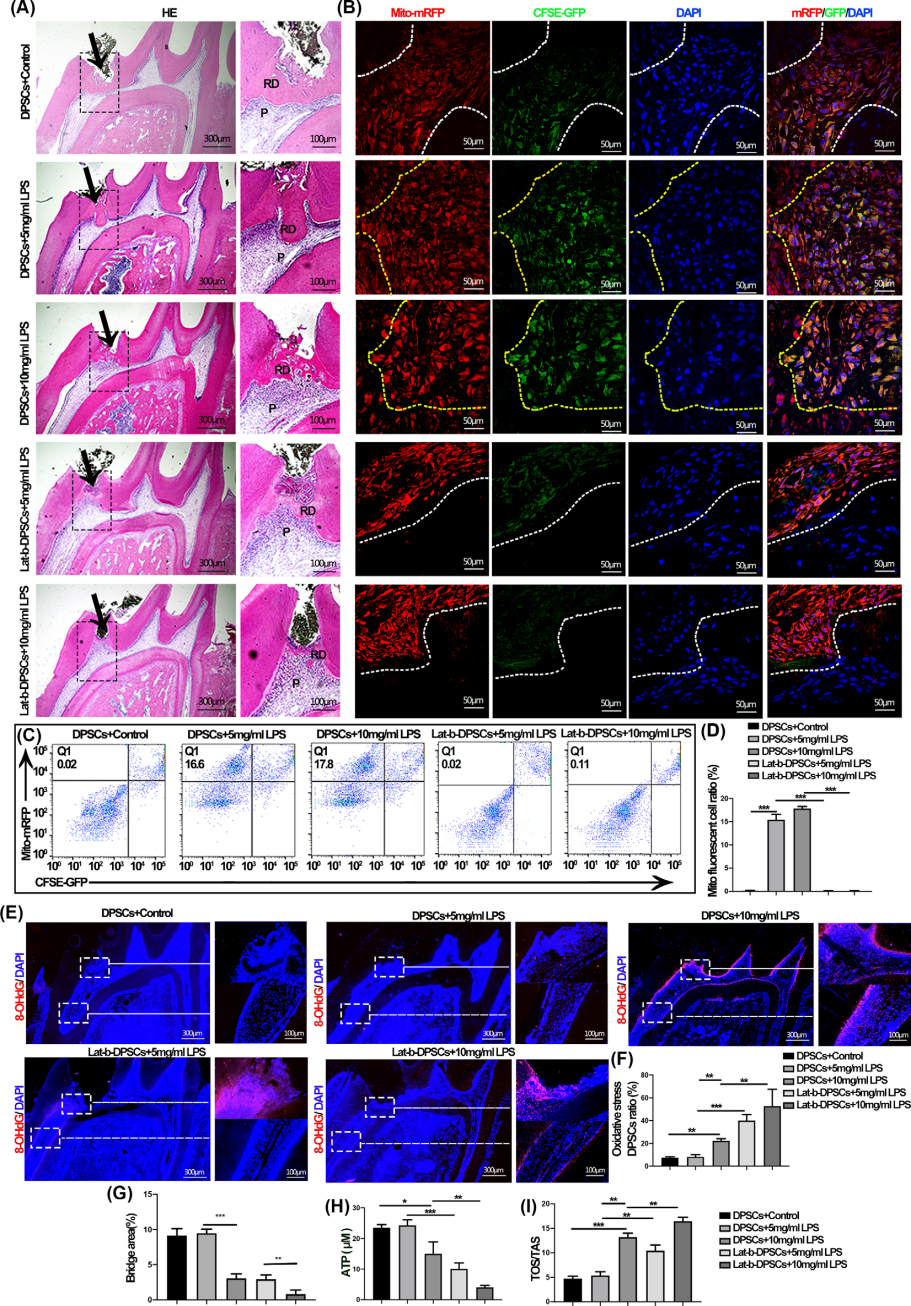
Mitochondrial transfer promotes the pulp‐dentin complex generation. (A) HE staining results of the control, mitochondrial transfer and mitochondrial transfer inhibition groups. The higher magnification field of view is the part indicated by the black arrow in the lower magnification diagram, respectively. The yellow arrow indicates the location of the implanted rat dental pulp stromal cells (rDPSCs). RD, reparative dentin; P, pulp. (B) Mitochondria from healthy DPSCs (Mito‐mRFP and CFSE‐GFP) transferred into injured DPSCs after co‐culture for 24 h. The yellow dashed lines select the recipient cells where the mitochondrial transfer occurs, while the white dashed line indicates cells where mitochondrial transfer did not occur. (C) The proportion of monofluorescent DPSCs in dental pulp tissue by FACS. (D) Quantitative analysis of the shift of mitochondrial red fluorescence among groups in vivo. (E) 8‐OHdG immunofluorescence staining of DPSCs at the proximal and distal end of the injury site, the right panel shows the higher magnification field of view in the white box in the left panel. (F) The value for DPSCs experiencing oxidative stress among groups. (G) ATP content in pulp tissue among groups. (H) TOS/TAS in the dental pulp tissue of the control group, the mitochondrial transfer group, and the mitochondrial transfer inhibition group. (*p < 0.05, **p < 0.01, ***p < 0.001, *n* = 5).

By constructing DPSCs injury models in vivo and in vitro, the results showed that dental pulp injury leads to OS, abnormal mitochondrial structure and function, and abnormal inter‐organelle contact structures and cellular function impairment in DPSCs (*p* < 0.001; Figure S1). We attempted to restore the mitochondrial function of injured DPSCs by adding healthy DPSCs and promoting mitochondrial transfer. Healthy rat DPSCs (rDPSCs) were extracted and implanted into the site of pulpal injury after labelling with mito‐mRFP and CFSE‐GFP. The results showed an apparent mitochondrial transfer and a significant increase in reparative dentin formation after adding DPSCs in the 5 mg/mL group and 10 mg/mL group (*p* < 0.001; Figure 1A–D, G). Nevertheless, the reparative dentin of the 10 mg/mL group still could not form complete calcified bridges. In contrast, the pro‐injury repair effect and mitochondrial transfer of DPSCs were significantly inhibited by adding mitochondrial transfer inhibitor (Lat‐b) pre‐treated rDPSCs. Meanwhile, we found that mitochondrial transfer had a reparative effect on apoptosis and inflammation in DPSCs (Figure S3A). The above results imply that the addition of exogenous DPSCs can exert mitochondrial transfer function to repair pulp tissue and ultimately promote pulp‐dentin complex repair. Further studies revealed that mitochondrial transfer of DPSCs could repair OS and mitochondrial dysfunction in injured DPSCs (*p* < 0.001; Figure 1E–I).

### 3.2 Mitochondrial transfer promotes functional recovery of injured DPSCs

**FIGURE 2:**
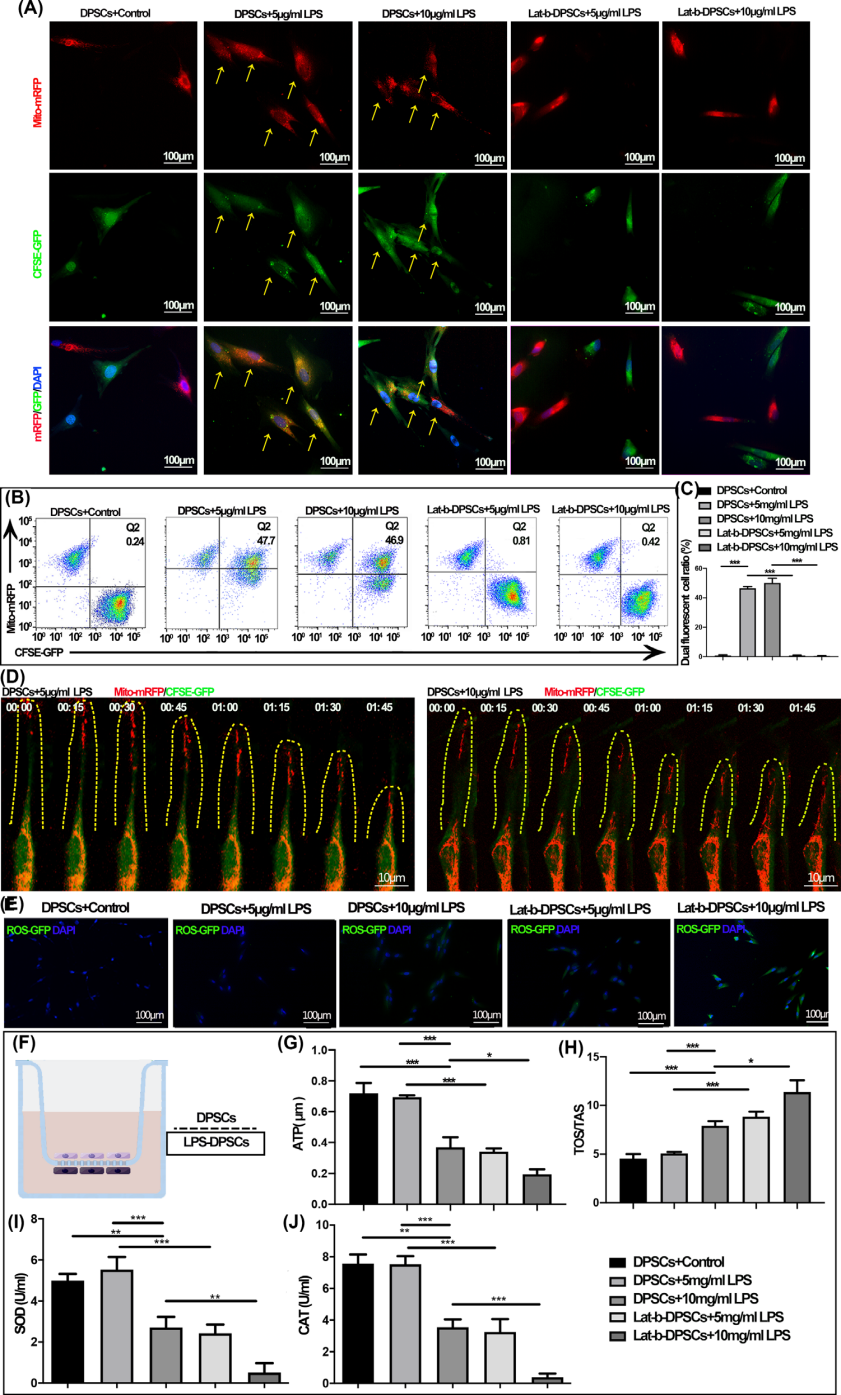
Mitochondrial transfer affected the function of injured dental pulp stromal cells (DPSCs). (A) Mitochondria from healthy DPSCs (Mito‐mRFP) transferred injured DPSCs (CFSE‐GFP) after co‐culture for 24 h. The yellow arrows indicate the recipient cell where the mitochondrial transfer occurs. (B) FACS shows a dual fluorescent cell ratio after co‐culture for 24 h. (C) Quantitative analysis of the shift of mitochondrial red fluorescence to green fluorescence among groups in vitro. (D) The dynamic movement of mito‐mRFP‐labelled mitochondria between DPSCs using confocal live‐cell images. The yellow dashed line marks the transferred mitochondria. (E) Reactive oxygen species fluorescence results of DPSCs. (F) Flow chart for studying injured DPSCs by indirect co‐culture (G) Changes in ATP content after mitochondrial transfer. (H) TOS/TAS levels after mitochondrial transfer of DPSCs. (I) SOD activity of DPSCs after mitochondrial transfer. (J) CAT activity of DPSCs after mitochondrial transfer (*p < 0.05, **p < 0.01, ***p < 0.001, *n* = 5).

To further determine the occurrence of mitochondrial transfer between DPSCs in vitro, we treated DPSCs stimulated with different LPS concentrations as mitochondrial transfer recipients. The healthy DPSCs were co‐cultured with recipient‐injured DPSCs for 24 h. The recipient DPSCs were stained with CFSE‐GFP, and the donor DPSCs were stained with mito‐mRFP before co‐culture. The result showed that, consistent with in vivo results when recipient DPSCs show significant OS and mitochondrial dysfunction; there was an apparent mitochondrial transfer phenomenon between DPSCs (*p* < 0.001; Figure 2A–C). Live cell imaging also showed that mitochondrial transfer occurred. Donor cell mitochondria dynamically migrated toward the adjacent DPSCs, as demonstrated by the colocalization of mitochondria with the recipient cells due to fluorescence (Figure 2D and Movies S1 and S2). We then sought to verify the restoration effect of mitochondrial transfer on the function of injured DPSCs in vitro. A three‐dimensional transwell culture system was established to separate injured DPSCs after co‐culture (Figure 2F). The mitochondrial membrane potential and intracellular ROS content also recovered, indicating that mitochondrial transfer significantly restores the mitochondrial function of injured DPSCs (Figures 2E and S3B). The ATP content, antioxidant levels, SOD and CAT activities of injured DPSCs were also improved after mitochondrial transfer (*p* < 0.001; Figure 2G–J).

Further, the mitochondrial transfer also regulates mitochondrial structure and proliferation and differentiation (*p* < 0.001; Figure S3C–F). Nevertheless, consistent with the in vivo results, the efficiency of mitochondrial transfer was not further enhanced when the injury was more severe. We then stimulate healthy cells with low concentrations of LPS to observe the impact on the efficiency of mitochondrial transfer. We found that the efficiency of mitochondrial transfer increased significantly when the healthy cells were stimulated with LPS at a concentration of 1 μg/mL and decreased in the 5 μg/mL LPS group (*p* < 0.001; Figure S4A–C). At the same time, we also found that stimulation of donor cells with low concentrations of LPS did not significantly promote the mitochondrial transfer injury repair effect but even inhibited it to some extent (*p* < 0.001; Figure S4D–I).

### 3.3 ER co‐transfers with mitochondria and regulates mitochondrial motility

**FIGURE 3:**
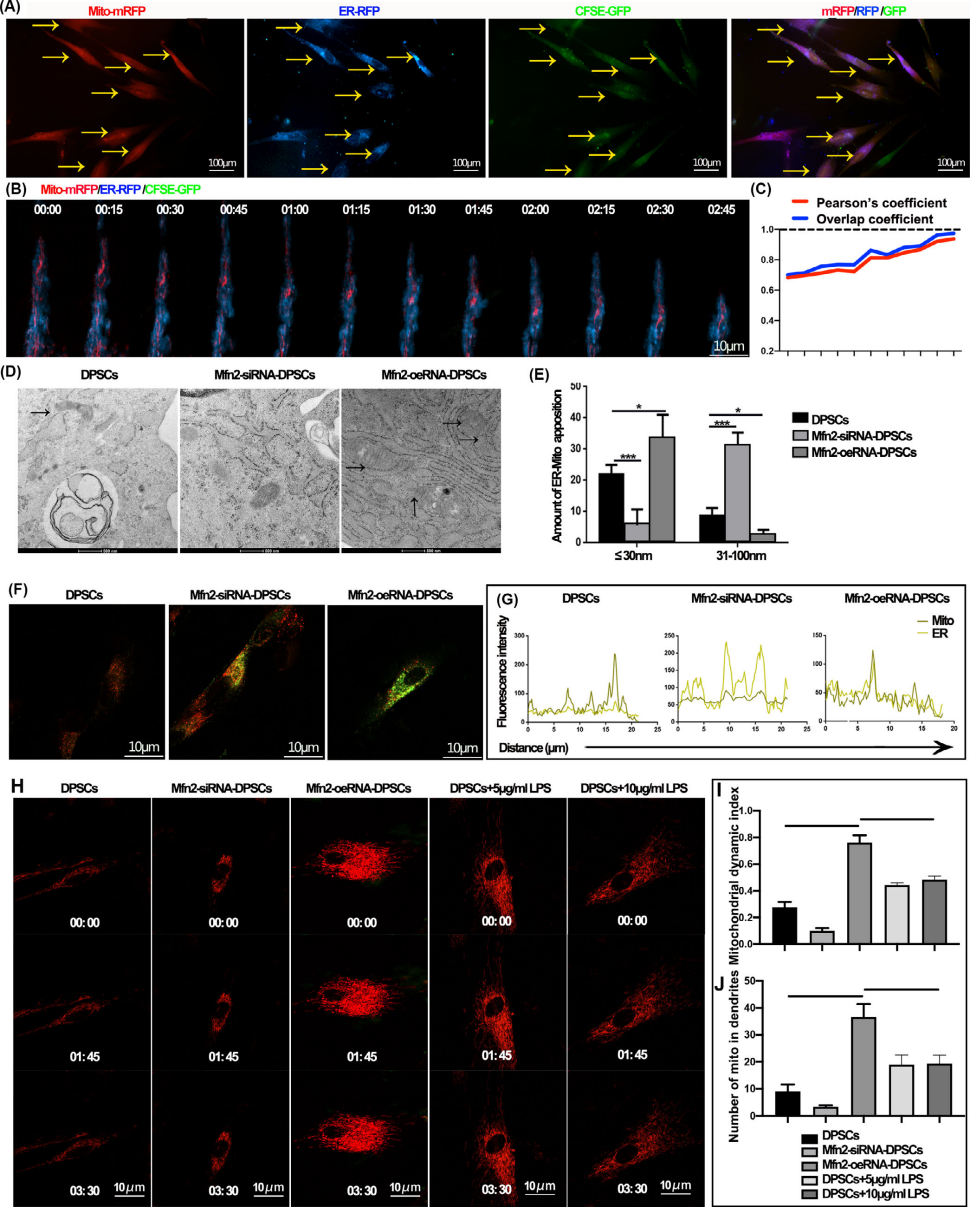
Endoplasmic reticulum (ER)‐mitochondrial contact regulates mitochondrial motility. (A) The mitochondrial‐ER transfer was observed through metastasis of mitochondrial red and ER blue to green fluorescence after co‐culture for 24 h. The yellow arrow indicates mitochondria‐ER co‐transfer dental pulp stromal cells (DPSCs). (B) Confocal time‐lapse images show that the mitochondria‐ER co‐transfer toward the adjacent recipient cell. (C) Overlap and Pearson's coefficient analysis show the dynamic association between ER and mitochondria. (D) Transmission electron microscopy of cellular mitochondria and ER structures, and inter‐organelle linkage structures after modulation of Mfn2 expression, the black arrows identify the mitochondria‐ER contact. (E) Quantitative analysis of mitochondria‐ER contacts. (F, G) Intracellular ER‐mitochondrial colocalization after modulation of Mfn2 expression. (H) The dynamic movement of mito‐mRFP‐labelled mitochondria of healthy DPSCs after mitochondrial transfer. (I) Assessment of mitochondrial dynamics. (J) Quantitative analysis of mitochondria in the dendritic structure of DPSCs (*p < 0.05, **p < 0.01, ***p < 0.001, *n* = 5).

The close association between mitochondria and ER underscores the importance of structural and functional interactions required for cellular homeostasis. This suggests that mitochondrial‐ER co‐transfer has a critical regulatory effect on mitochondrial transfer efficiency. Reviewing the literature on mitochondrial transfer, we found a co‐transfer of mitochondria‐ER in osteoblasts.^24^ By simultaneously labelling donor cell mitochondria and ER, we examined whether ER regulates mitochondrial transfer or modulates the functional reparative effects of mitochondrial transfer. We observed the colocalization of mitochondria with ER within the recipient cells (Figure 3A). Further, live cell imaging showed that mitochondria transfer within dendrites was dynamically colocalized with ER (Figure 3B,C and Movie S3). We then tried to block or promote inter‐organelle binding by lentiviral silencing or overexpression of Mfn2 in donor DPSCs. Modulation of Mfn2 expression had minimal effect on ATP synthesis function and mitochondrial membrane potential but significantly altered ER‐mitochondrial colocalization and contact distance (*p* < 0.001; Figures S5 and 3D–G). Mfn2 showed significant alterations in mitochondrial network distribution, and the knockdown of Mfn2 significantly reduced mitochondrial distribution within the dendrites of DPSCs and mitochondrial motility (*p* < 0.001; Figure 3H–J, and Movies [Link], [Link]).

### 3.4 ER‐mitochondrial contact regulates the ER‐mitochondrial co‐transfer of DPSCs

**FIGURE 4:**
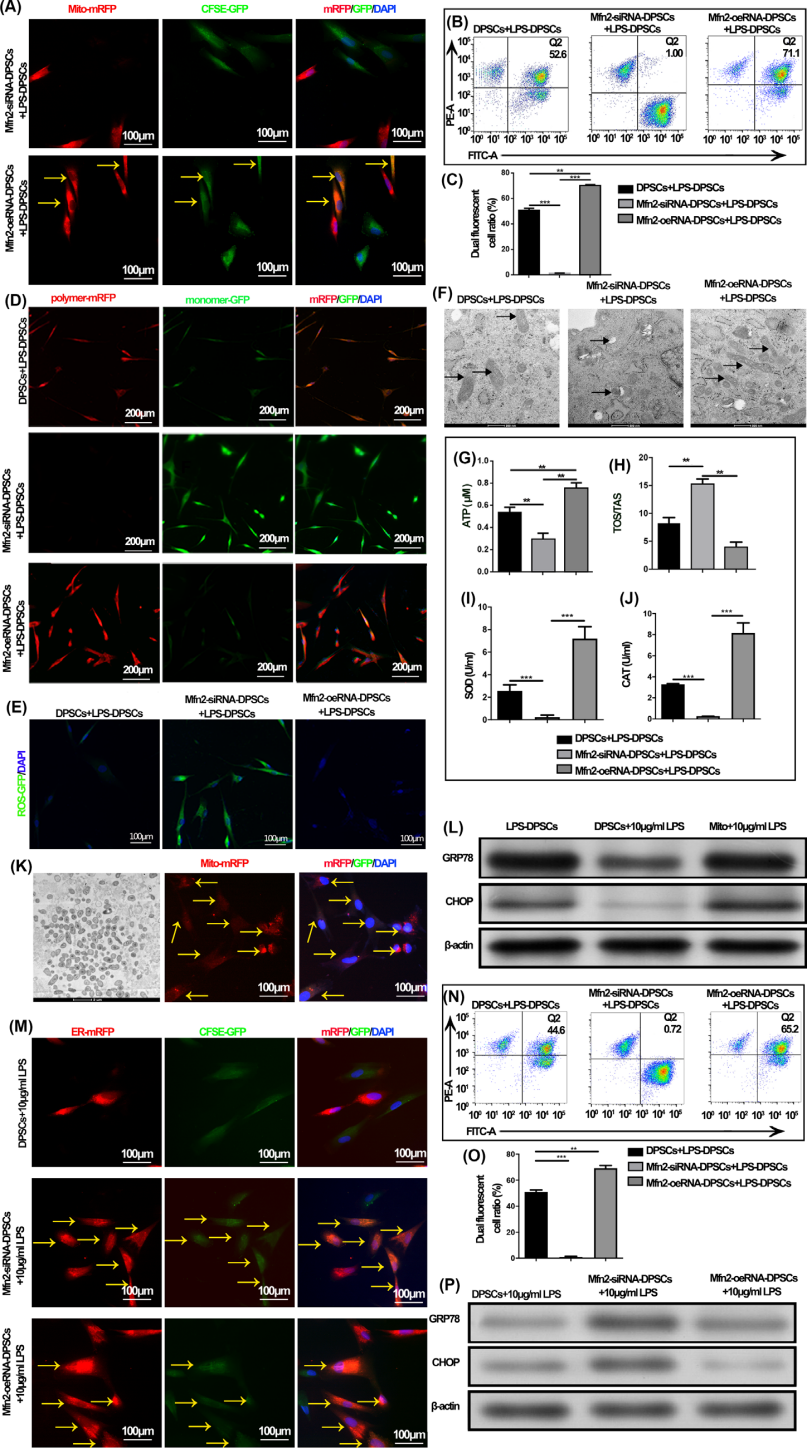
The regulation of mitochondrial transfer promotes the functional recovery of injured dental pulp stromal cells (DPSCs). (A) Mitochondria from healthy DPSCs (Mito‐mRFP) transferred into pre‐stained CFSE‐GFP injured DPSCs after co‐culture for 24 h. The yellow arrows indicate the recipient cell where the mitochondrial transfer occurs. (B) FACS shows dual fluorescent cell ratios after modulation of Mfn2. (C) Statistical analysis of the double fluorescent cell ratio. (D) Observation of mitochondria membrane potential in recipient cells after endoplasmic reticulum (ER) regulation of mitochondrial transfer. (E) Reactive oxygen species fluorescence results of DPSCs after ER regulation of mitochondrial transfer. (F) Transmission electron microscopy from mitochondria of DPSCs. (G) Changes in ATP content in recipient cells after ER regulation of mitochondrial transfer. (H) TOS/TAS levels change after ER regulation of mitochondrial transfer. (I) SOD activity of DPSCs after ER regulation of mitochondrial transfer. (J) CAT activity of DPSCs after ER regulation of mitochondrial transfer. (K) Transmission electron microscopy results of mitochondria extracted from DPSCs and the fluorescence result of mitochondrial transplantation. (L) WB results of ERS related proteins GRP78 and CHOP. (M) ER from healthy DPSCs (Mito‐mRFP) was transferred into pre‐stained CFSE‐GFP injured DPSCs after co‐culture for 24 h. The yellow arrows indicate the recipient cell where the ER transfer occurs. (N) FACS shows dual fluorescent cell ratios after modulation of Mfn2. (O) Statistical analysis of the double fluorescent cell ratio. (P) WB results of ERS‐related proteins GRP78 and CHOP after modulation of Mfn2 (*p < 0.05, **p < 0.01, ***p < 0.001, *n* = 5).

Further investigation found that modulation of mitochondrial‐ER binding significantly affected the mitochondrial transfer efficiency of DPSCs (*p* < 0.001; Figure 4A–C). Overexpression of Mfn2 increases mitochondrial‐ER contacts and promotes mitochondrial transfer, whereas inhibition of Mfn2 results in a substantial loss of mitochondrial transfer. Furthermore, donor cells lacking ER‐mitochondrial contacts could not restore mitochondrial metabolic functions and cellular functions of injured DPSCs, such as mitochondrial membrane potential, ATP production, OS and cell proliferation and differentiation functions. The above results show that ER affects the mitochondrial transfer of DPSCs in vitro and their mitochondrial function and cellular function restoration effects. Moreover, siRNA‐Mfn2 treated DPSCs that lacked sufficient ER‐mitochondria contacts were not able to restore metabolic function when co‐cultured with injured DPSCs, as indicated by ROS and the levels of ATP, TOS/TAS, SOD, and CAT activity (*p* < 0.001; Figure 4D–J). In contrast, overexpression of Mfn2 significantly increased the mitochondrial transfer rate, and more injured DPSCs could receive mitochondria and significantly restore their mitochondrial function. These results suggest ER mediates mitochondrial transfer between adjacent DPSCs to synchronize their energy‐consuming metabolism and Mfn2 is a critical participant. ER from donor DPSCs may bind to mitochondria to initiate the mitochondrial transfer.

Moreover, we further illustrated that ER‐mitochondrial co‐transfer to injured DPSCs affects ER function and ER stress in injured cells. First, we found that ERS also accompanied pulp injury and functional impairment of DPSCs. Combined with the literature, ERS may also be involved in pulp injury repair (Figure S6). Next, by extracting mitochondria and transplanting them into recipient cells, we confirmed that mitochondrial transplantation having essentially no beneficial impact on the ERS of injured cells due to the absence of ER involvement, suggesting a role for ER after transfer to injured cells (Figure 4K,L). Through Mfn2 regulation of mitochondrial transfer, we found that ER transfer was also regulated in parallel, which further suggested ER‐mitochondrial co‐transfer (*p* < 0.001; Figure 4M–O). Also, the modulation of ER‐mitochondrial co‐transfer has a significant modulatory effect on ERS in injured cells (Figure 4P).

### 3.5 Mfn2 promotes ER‐mitochondrial co‐transfer for pulp injury repair

**FIGURE 5:**
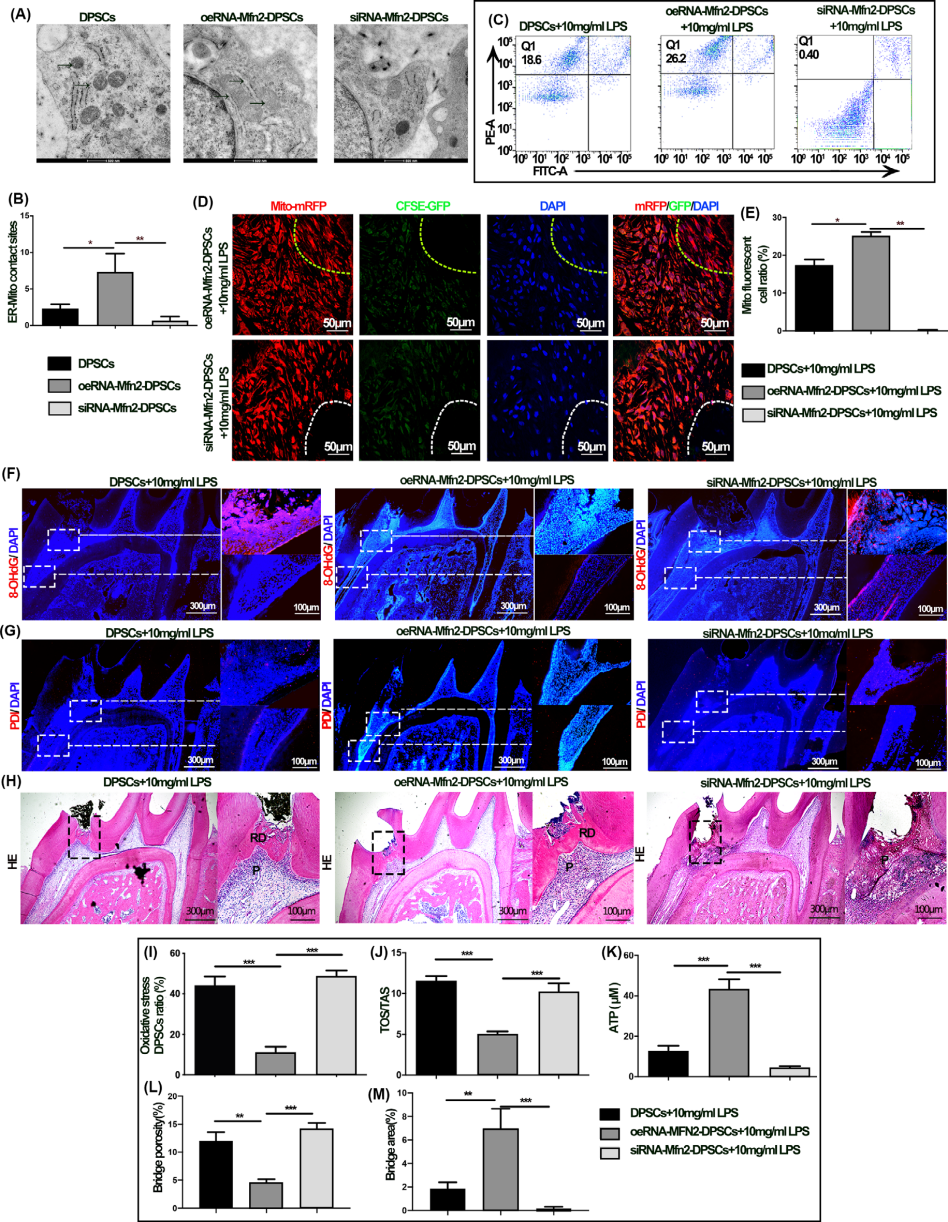
Endoplasmic reticulum (ER)‐mitochondrial co‐transfer promotes pulpal injury repair and pulp‐dentin complex generation. (A) Transmission electron microscopy results of rat dental pulp stromal cells (rDPSCs) mitochondria. (B) Statistical results on the number of mitochondria‐ER contacts. (C) The proportion of monofluorescent and fluorescent cells in dental pulp tissue by FACS after regulating the expression of the Mfn2 in donor DPSCs. (D) Mitochondria from healthy DPSCs (Mito‐mRFP and CFSE‐GFP) transferred into injured DPSCs after co‐culture for 24 h. The yellow dashed lines select the recipient cells where the mitochondrial transfer occurs, while the white dashed line indicates cells where mitochondrial transfer did not occur. (E) Statistical results of flow cytometric analysis. (F) 8‐OHdG immunofluorescence staining of DPSCs at the proximal and distal end of the injury site, the right panel shows the higher magnification field of view in the white box in the left panel. (G) The right panel of PDI immunofluorescence staining of DPSCs at the proximal and distal end of the injury site shows the higher magnification field of view in the white box in the left panel. (H) HE staining results after regulating the expression of the Mfn2 proteins in donor DPSCs. The black arrow in the low magnification diagram indicates the high magnification field of view. P, pulp; RD, reparative dentin. (I) Statistical analysis of the percentage of oxidative stress DPSCs. (J) Statistical analysis of TOS/TAS levels of DPSCs. (K) ATP content of dental pulp tissue after regulating the expression of the Mfn2 proteins in healthy DPSCs. (L, M) Quantification of dentin bridge porosity and area of reparative dentin (*p < 0.05, **p < 0.01, ***p < 0.001, *n* = 5).

The last step of our work was to modulate Mfn2 of rDPSC to validate in vivo mitochondrial transfer and its function. First, we found that Mfn2 regulates mitochondrial‐ER contacts in rDPSCs and regulates mitochondrial transfer in the dental pulp (*p* < 0.001; Figure 5A–E). The alteration of mitochondrial transfer efficiency further alters the reparative dentin formation, the degree of OS in the pulp tissue is reduced, and the mitochondrial function and ERS are restored (*p* < 0.001; Figure 5F–K). In turn, with the alteration of mitochondrial transfer efficiency, the formation and structure of healthy dentin were changed (*p* < 0.001; Figure 5L–M), and the proliferation and differentiation functions of DPSCs were altered (*p* < 0.001; Figure S7). The above results again verified the repairing effect of mitochondrial transfer on pulpal injury and the regulation of mitochondrial transfer function by ER.

## 4 DISCUSSION

The most typical features of the dental pulp environment are malnutrition and hypoxia,^22^, ^29^, ^30^ studies have shown that the oxygen content in the pulp cavity is about 5%, which is much lower than the normal oxygen concentration of 21%, and the microenvironmental oxygen concentration is even as low as 1% due to the fully developed apical foramen of the mature pulp without sufficient blood supply. These conditions will lead to more severe OS and mitochondrial function of DPSCs after pulp injury. Current strategies for pulpal injury repair are mainly through the addition of growth factors and implantation of DPSCs. While exogenous growth factors are currently difficult to effectively address the mitochondrial dysfunction caused by hypoxia, long‐term survival of transplanted healthy DPSCs is also difficult due to the low nutrient supply and oxygen. For the first time, we identified mitochondrial transfer of DPSCs in vivo and in vitro models and found that mitochondrial transfer played a role in promoting pulpal injury repair. This may be a feasible therapeutic strategy for applying DPSCs, because mitochondrial transfer occurs early in severe injury and rapidly and can effectively restore mitochondrial dysfunction. Using a three‐dimensional transwell co‐culture model, we observed that healthy DPSCs could restore mitochondrial function in injured DPSCs. ATP, OS, ROS, and mitochondrial membrane potential were restored in injured DPSCs, indicating the functional significance of mitochondrial transfer between DPSCs. We also found that pulp injury can lead to ROS accumulation and OS by affecting the activity of CAT and SOD. In turn, OS and cell death are closely linked and can cause apoptosis and necrosis. Our study shows that mitochondrial transfer can restore OS in the dental pulp caused by injury and restore cellular activity and function. These findings are consistent with other studies, showing that mitochondrial transfer is crucial for tissue repair.^30^, ^31^, ^32^ All of the above evidence indicates the critical role of mitochondrial transfer for pulp injury repair and provides a new direction for the study of living pulp preservation.

To find the regulatory factors of mitochondrial transfer, we observed the mitochondrial transfer between DPSCs by live cell imaging and found that the degree of acceptor cell injury did not alter the motility and dynamic of donor‐derived mitochondria. Recent studies addressing the mechanism of mitochondrial transfer have found that it is activated by injury‐associated molecular pattern molecules (DAMPs) released from receptor cells.^33^, ^34^, ^35^, ^36^ However, our study found that, although injury to DPSCs can trigger mitochondrial transfer, different degrees of injury may not further affect the rate and efficiency of mitochondrial transfer. As for the main factors that regulate mitochondrial motility in donor cells and improve the efficiency of mitochondrial transfer, we found that ER may play a major role. Via live cell imaging, we further observed ER‐mitochondrial co‐transfer. By modulating Mfn2 to affect ER‐mitochondrial binding, mitochondrial transfer was also significantly affected. Dynamic interactions between mitochondria and other organelles, particularly the ER, are essential for mitochondrial motility.^37^, ^38^ In a study of rat dorsal root ganglion neurons, by modulating Mfn2, researchers observed reduced mitochondrial motility in distal axonal regions, further corroborating the role of ER‐mitochondrial binding in regulating mitochondrial transfer.^39^ Also, the mitochondrial‐ER co‐transfer was found in a mitochondrial transfer study of osteoblasts.^24^ Additionally, we found the necessity of mitochondrial‐ER binding for mitochondrial transfer. Free mitochondria do not seem to be released through TNTs. And we also found that by enhancing mitochondria‐ER binding, the efficiency of mitochondrial transfer was also significantly enhanced. Studies have concluded that organelles move along microtubules to establish and maintain their proper distribution and function, the ER regulates the distribution of organelles on microtubules, and when ER localization on microtubules is disrupted, the distribution of other organelles is also affected.^40^ All these results further corroborate the importance of mitochondrial‐ER contact for mitochondrial transfer.

Simultaneously, our results suggest that ER regulates mitochondrial transfer and may have regulatory effects on ER function and ERS in recipient DPSCs. ER is responsible for synthesizing, processing, and assembly of secreted and membrane proteins. It also acts as a quality control agent by removing incorrectly folded intermediates through ER‐related degradation systems.^41^, ^42^ Consequently, ER is essential for maintaining cellular functional homeostasis. Under various stress conditions, such as an imbalance of intracellular calcium homeostasis, OS, and infection, ER can become dysfunctional and disrupted, thus inducing ERS.^43^, ^44^ ERS can lead to the accumulation of unfolded or misfolded proteins, thereby yielding unfolded protein response ER overload response and apoptosis.^45^, ^46^, ^47^, ^48^ Our study is the first to highlight the phenomenon of ERS in dental pulp injury, and we have initially proposed the recovery of ERS in injured cells after ER transfer. However, the mode and mechanism of action of ER after entering recipient cells are urgently required. The method and agency of action of ER after entering recipient cells must be further investigated. Whether mitochondria are in contact with other organelles, such as lysosomes, and whether such contact could be a regulatory factor for mitochondrial transfer also deserve further investigation. Moreover, We found that Mfn2 not only affects the binding of mitochondria and ER, but its alteration of Mfn1 and Opa‐1 also indicates that mitochondrial fusion is altered, which led us to further think about the effect of mitochondrial fusion and division on mitochondrial transfer.

Our findings demonstrate that mitochondria are dynamically transferred between the DPSCs. Healthy DPSCs can transfer mitochondria to mitochondrial dysfunction DPSCs and reinstates OS, ATP levels, mitochondrial membrane potential and reduces ROS accumulation. Furthermore, we found that by regulating ER‐mitochondrial contacts, DPSCs mitochondrial motility and transfer can be regulated. Ultimately, we discovered that mitochondrial–ER co‐transfer could play a role in injury repair and regulate mitochondrial function and ERS in injured cells.

## AUTHOR CONTRIBUTIONS

Qi Zhang conceived the project. Xiaoyi Zhang prepared all the materials, conducted the in vitro experiments, and analysed the samples of in vivo experiment. Chunmeng Wang established the animal model and performed the in vivo experiments. Zihao Zhou performed the in vitro experiments. All authors discussed the results and commented. Xiaoyi Zhang completed the first draft and drew the schematic figures. All authors modified, and Qi Zhang finalized the manuscript.

## CONFLICT OF INTEREST STATEMENT

The authors declare no conflict of interest.

## Supporting information


**Data S1.** Supporting Information.

**Movie S1.** Healthy DPSCs mitochondria (mito‐mRFP, Red) dynamically move to 5 μg/mL LPS‐DPSCs (CFSE‐GFP, Green).

**Movie S2.** Healthy DPSCs mitochondria (mito‐mRFP, Red) dynamically move to 10 μg/mL LPS‐DPSCs (CFSE‐GFP, Green).

**Movie S3.** Dynamic movement of healthy DPSCs mitochondria (mito‐mRFP, Red) and endoplasmic reticulum (ER‐RFP, Blue) to LPS‐DPSCs (CFSE‐GFP, Green).

**Movie S4.** Mitochondrial motility of DPSCs.

**Movie S5.** Mitochondrial motility of Mfn2‐siRNA‐DPSCs.

**Movie S6.** Mitochondrial motility of Mfn2‐oeRNA‐DPSCs.

**Movie S7.** Mitochondrial motility of mitochondrial transfer donor DPSCs(5 μg/mL LPS).

**Movie S8.** Mitochondrial motility of mitochondrial transfer donor DPSCs(10 μg/mL LPS).

## Data Availability

Some or all data supporting this study's findings are available from the corresponding author upon reasonable request.
