# Supplementary material for: The mitochondrial‐endoplasmic reticulum co‐transfer in dental pulp stromal cell promotes pulp injury repair
Source: Cell Prolif. 2023 Jul 26;57(1):e13530. doi: 10.1111/cpr.13530 (PMC10771100; doi:10.1111/cpr.13530)
Supplement: Supplementary file 1 — Data S1. Supporting Information. [file CPR-57-e13530-s005.docx]

**Appendix**

# The mitochondrial-endoplasmic reticulum co-transfer in dental pulp stromal cells promotes pulp injury repair

Xiaoyi Zhang^1^, Chunmeng Wan^1^, Zihao Zhou^1^and *Qi Zhang*^1§^

1Department of Endodontics, Stomatological Hospital and Dental School of Tongji University, Shanghai Engineering Research Center of Tooth Restoration and Regeneration, 200072, China., 399 Middle Yan Chang Road, Shanghai, 200072, China

#

^§^*Correspondence:*

*Qi Zhang*

*Address: No.399 Yanchang Middle Road, Jing'an District, Shanghai. Email: qizhang@tongji.edu.cn*

Table of Contests:

Figure S1. Pulp injury causes oxidative stress and mitochondrial dysfunction in DPSCs

Figure S2. Primary culture and identification of DPSCs.

Figure S3. The effects of mitochondrial transfer on the proliferation and differentiation function of DPSCs.

Figure S4. Mitochondrial transfer between DPSCs with different degrees of damage.

Figure S5. The Effect of Mfn2 on DPSCs mitochondrial function.

Figure S6. Pulp injury causes endoplasmic reticulum stress in DPSCs.

Figure S7. Mfn2 regulate the effects of mitochondrial transfer on DPSCs function.

Figure S8. Full-length gels of western blot.

Movie S1 (.mp4 format). Healthy DPSCs mitochondria (mito-mRFP, Red) dynamically move to 5 μg/ml LPS-DPSCs (CFSE-GFP, Green)

Movie S2 (.mp4 format). Healthy DPSCs mitochondria (mito-mRFP, Red) dynamically move to 10 μg/ml LPS-DPSCs (CFSE-GFP, Green)

Movie S3 (.mp4 format). Dynamic movement of healthy DPSCs mitochondria (mito-mRFP, Red) and endoplasmic reticulum (ER-RFP, Blue) to LPS-DPSCs (CFSE-GFP, Green)

Movie S4 (.mp4 format). Mitochondrial motility of DPSCs

Movie S5 (.mp4 format). Mitochondrial motility of Mfn2-siRNA-DPSCs

Movie S6 (.mp4 format). Mitochondrial motility of Mfn2-oeRNA-DPSCs

Movie S7 (.mp4 format). Mitochondrial motility of mitochondrial transfer donor DPSCs（5μg/ml LPS）

Movie S8 (.mp4 format). Mitochondrial motility of mitochondrial transfer donor DPSCs（10μg/ml LPS）


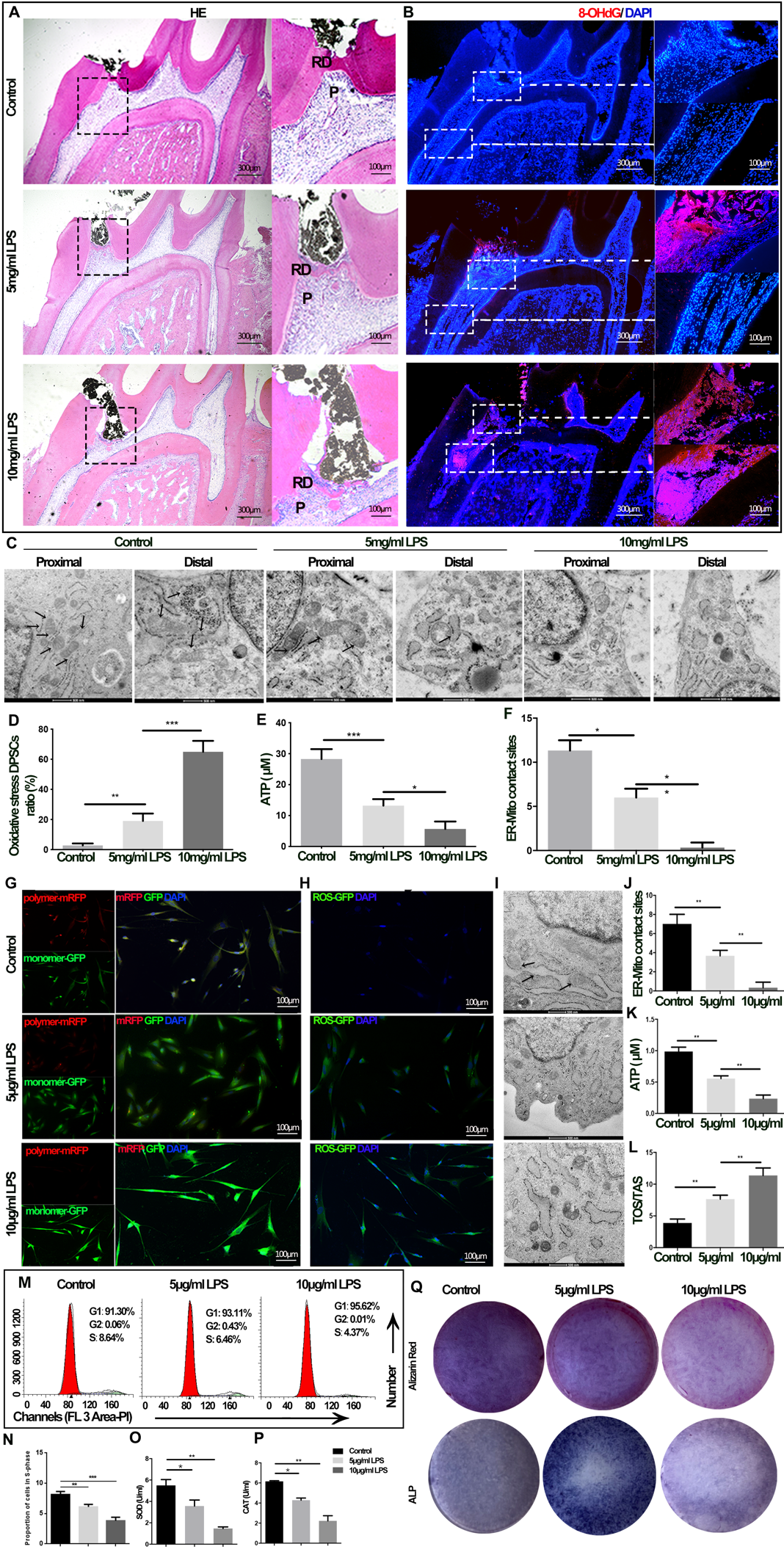


**Figure S1. Pulp injury causes oxidative stress and mitochondrial dysfunction in DPSCs**

(A) The HE staining results of LPS model of dental pulp injury in rat, the right panel shows the higher magnification field of view in the black box in the left panel, respectively. RD reparative dentin, P pulp. (B) 8-OHdG immunofluorescence staining of DPSCs at three days after pulp injury, the right panel shows the higher magnification field of view in the white box in the left panel. (C) Transmission electron microscopy of DPSCs in proximal and distal end of the injury site, the black arrows identify the mitochondria-ER contact. (D) The value for DPSCs experiencing oxidative stress at different injury levels. (E) ATP content in pulp tissue at 7 and 28 days after pulp injury. (F) Number of mitochondria-ER binding sites formed in coronal and root after three days of pulp injury. (G) Mitochondrial membrane potential of hDPSCs after culture in LPS for 24h. (H) Mitochondrial ROS of hDPSCs after culture in LPS for 24h. (I) Mitochondrial morphology of cells under transmission electron microscopy after culture in LPS, the black arrows identify the mitochondria-ER contact. (J) Mitochondria-ER binding sites after culture in LPS. (K) Cellular ATP content of hDPSCs at 24h after culture in LPS. (L) Total oxidant status/total antioxidant status (TOS/TAS) levels after culture in LPS for 24h. (M) Flow cytometric cycle analysis of DPSCs after LPS stimulation. (N) Statistical analysis of the flow cytometric cycle results of DPSCs after LPS stimulation. (O) SOD activity of DPSCs. (P) CAT activity of DPSCs. (Q) Alizarin red and ALP staining results after LPS stimulation. **p*< 0.05, ***p* < 0.01, ****p* < 0.001, n = 5.


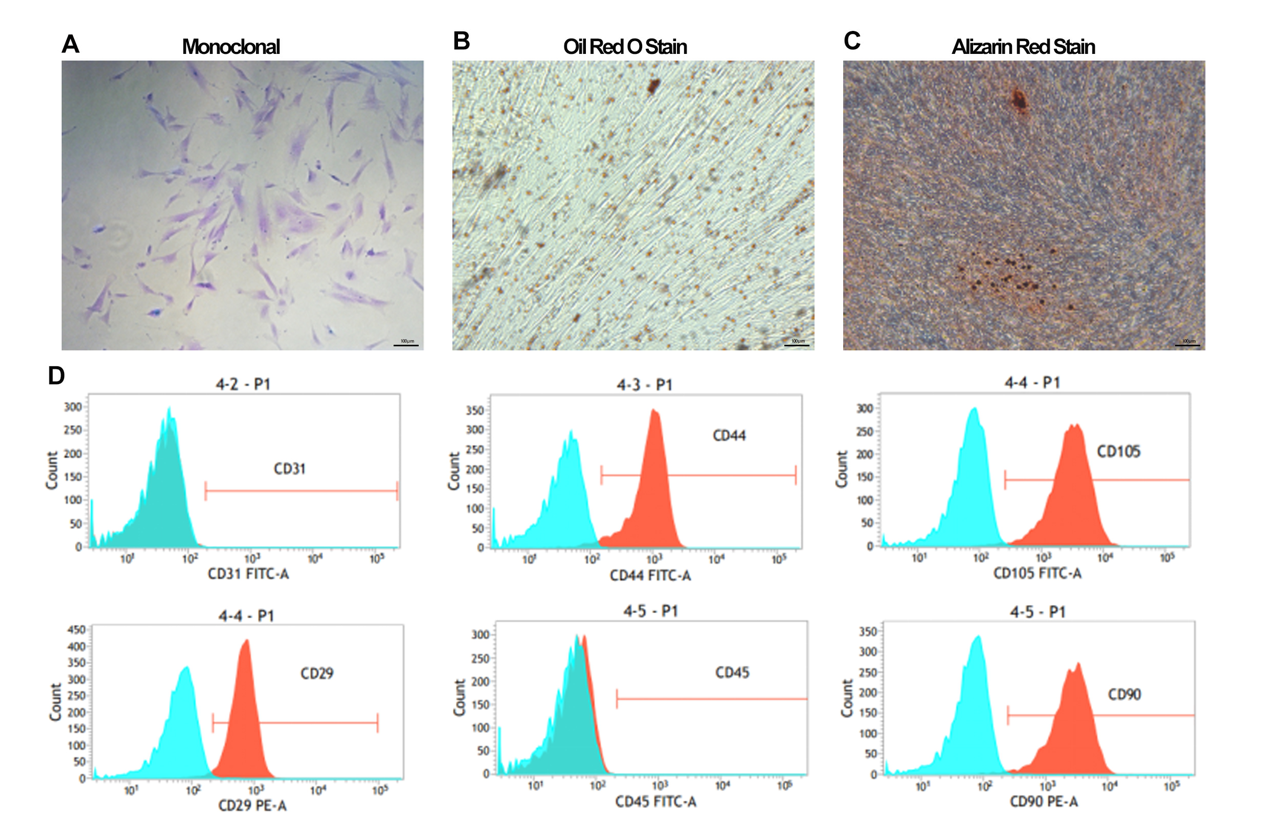


**Figure S2. Primary culture and identification of DPSCs.**

(A) Crystalline violet staining showing primary single-cell clones of DPSCs. (B) DPSCs Adipose differentiation. (C) DPSCs chondrogenic differentiation. (D) DPSCs surface marker identification. n = 5.


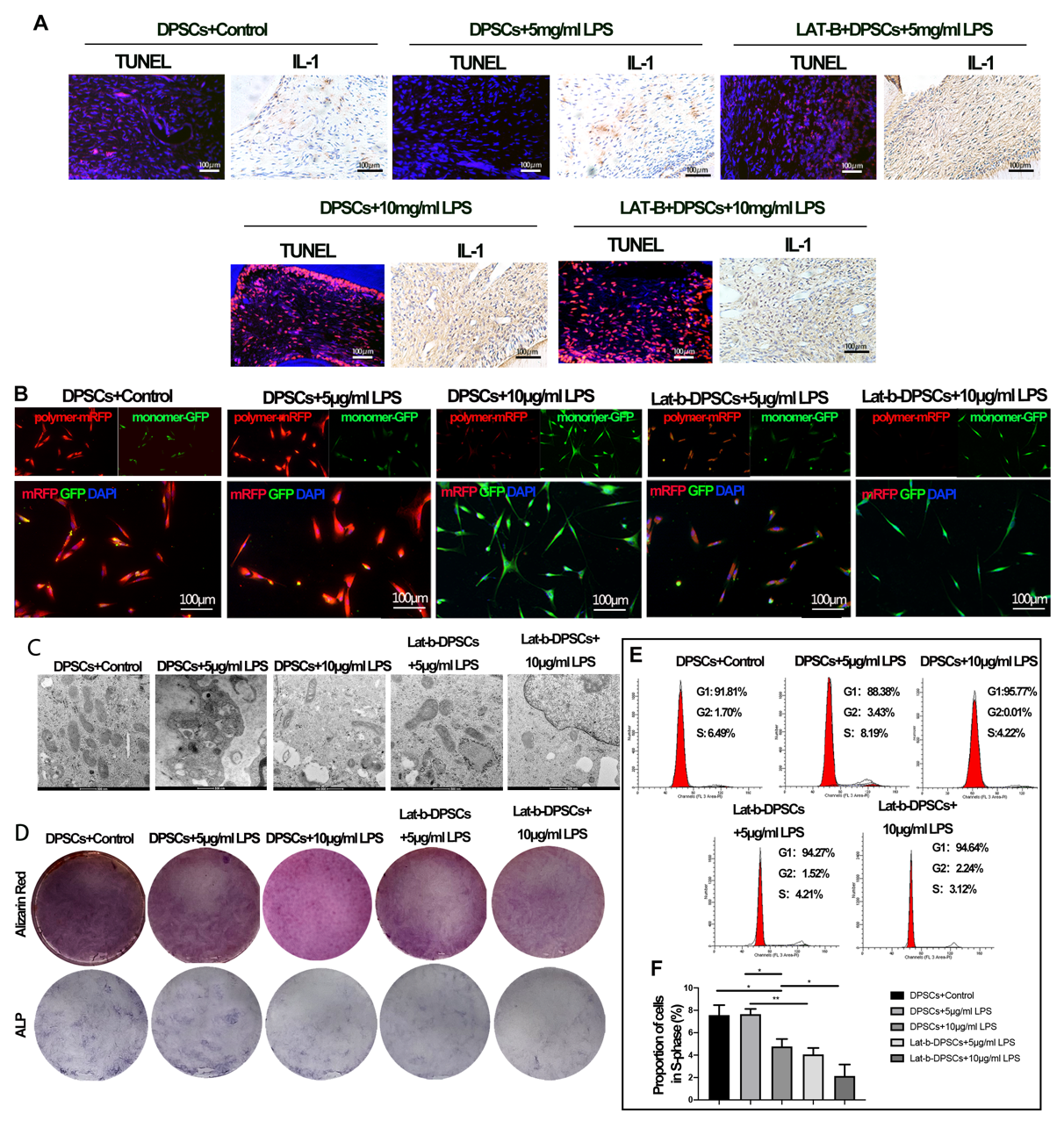


**Figure S3. The effects of mitochondrial transfer on the proliferation and differentiation function of DPSCs.**

(A) Results of TUNEL apoptosis staining and IL-1β immunohistochemistry after mitochondrial transfer. (B) Observation of mitochondria membrane potential in recipient cells. (C) Transmission electron microscopy from mitochondria of DPSCs. (D) Results of alizarin red and ALP staining of DPSCs after mitochondrial transfer. (E) Flow cytometric cycle results of DPSCs after mitochondrial transfer. (F) Statistical analysis of the flow cytometric cycle of DPSCs after mitochondrial transfer. **P* < 0.05, ***P*< 0.01, ****P* < 0.001, n = 5.


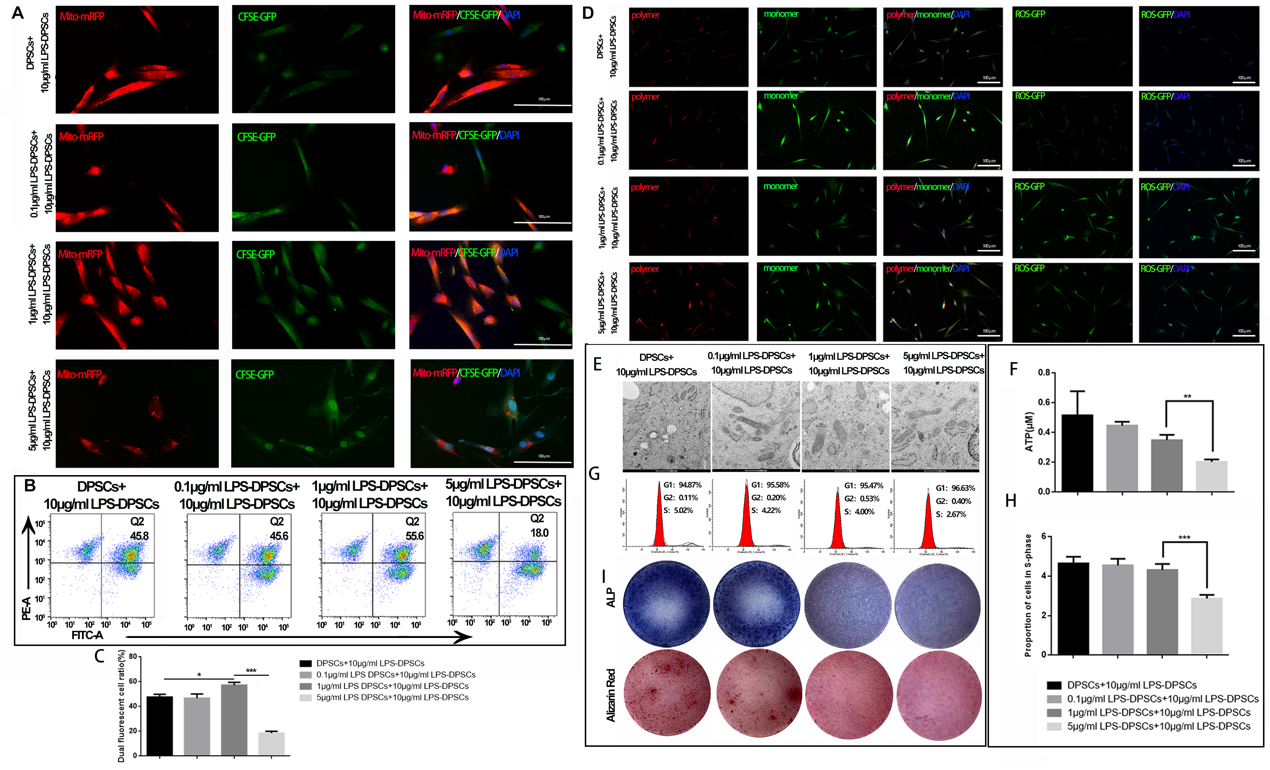


**Figure S4. Mitochondrial transfer between DPSCs with different degrees of damage.**

(A) Mitochondria were transferred from Mito- LPS-stimulated-DPSCs (in red) to CFSE-LPS-stimulated-DPSCs (in green) after co-culture for 24 h. (B) Flow cytometric analysis shows dual fluorescent cell ratio after co-culture for 24 h. (C) Quantitative statistical results of flow fluorescence analysis. (D) ROS generation and mitochondrial membrane potential changes in recipient cells after mitochondrial transfer. (E) Transmission electron microscopy from mitochondria of DPSCs. (F) ATP content of DPSCs after mitochondrial transfer. (G) Flow cytometric cycle results of DPSCs. (H) Statistical analysis of the DPSC flow cytometric cycle. (I) Results of Alizarin red and ALP staining of DPSCs. **p*< 0.05, ***p* < 0.01, ****p* < 0.001, n = 5.


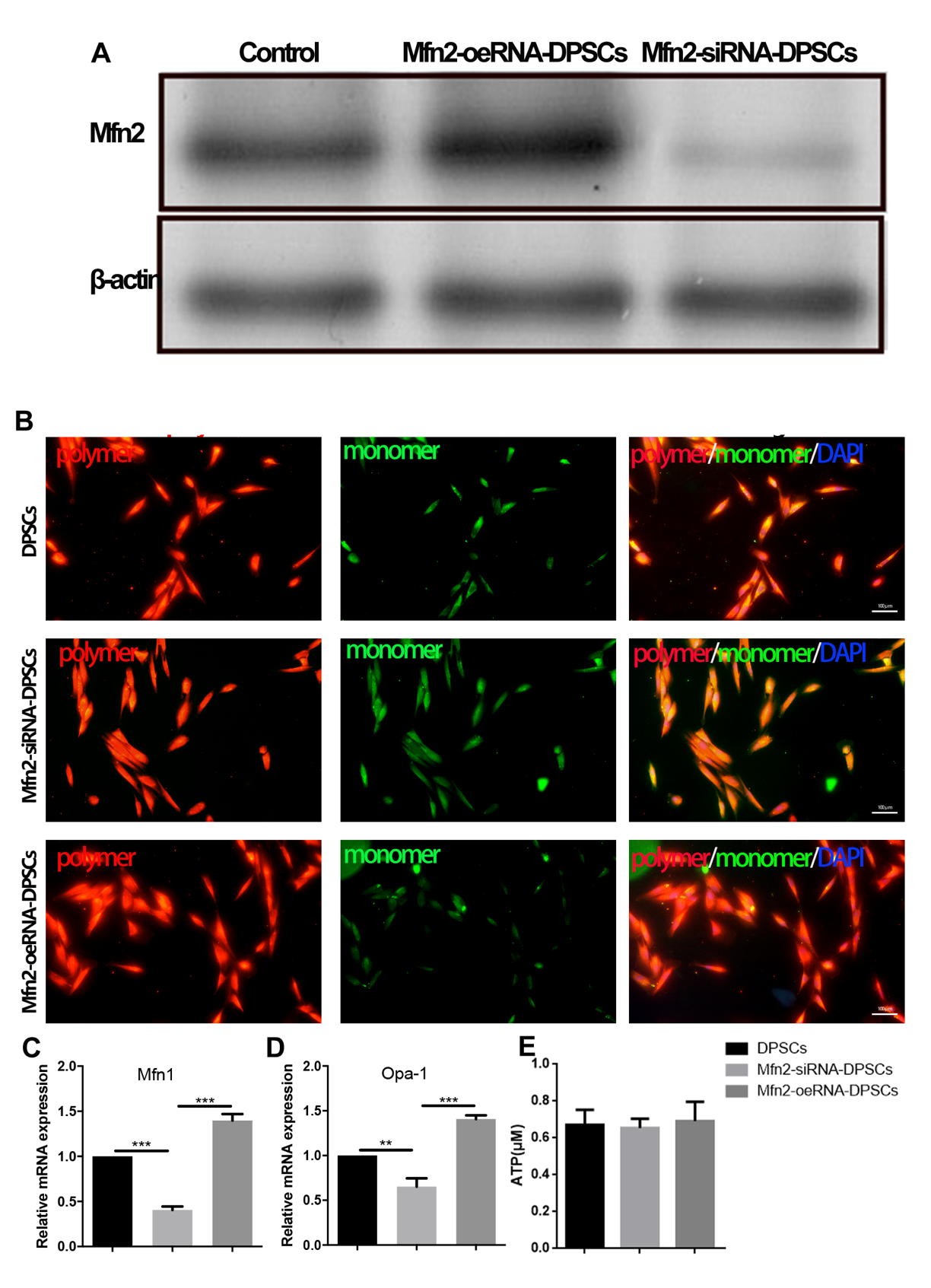


**Figure S5. The Effect of Mfn2 on DPSCs mitochondrial function.**

(A) Mfn2 protein expression after lentiviral transfection. (B) Mitochondrial membrane potential after lentiviral transfection. (C) Changes in Mfn1 mRNA after regulation of Mfn2. (D) Changes in Opa-1 mRNA after regulation of Mfn2. (E) ATP content after lentiviral transfection. **p* < 0.05, ***p* < 0.01, ****p* < 0.001, n = 5.


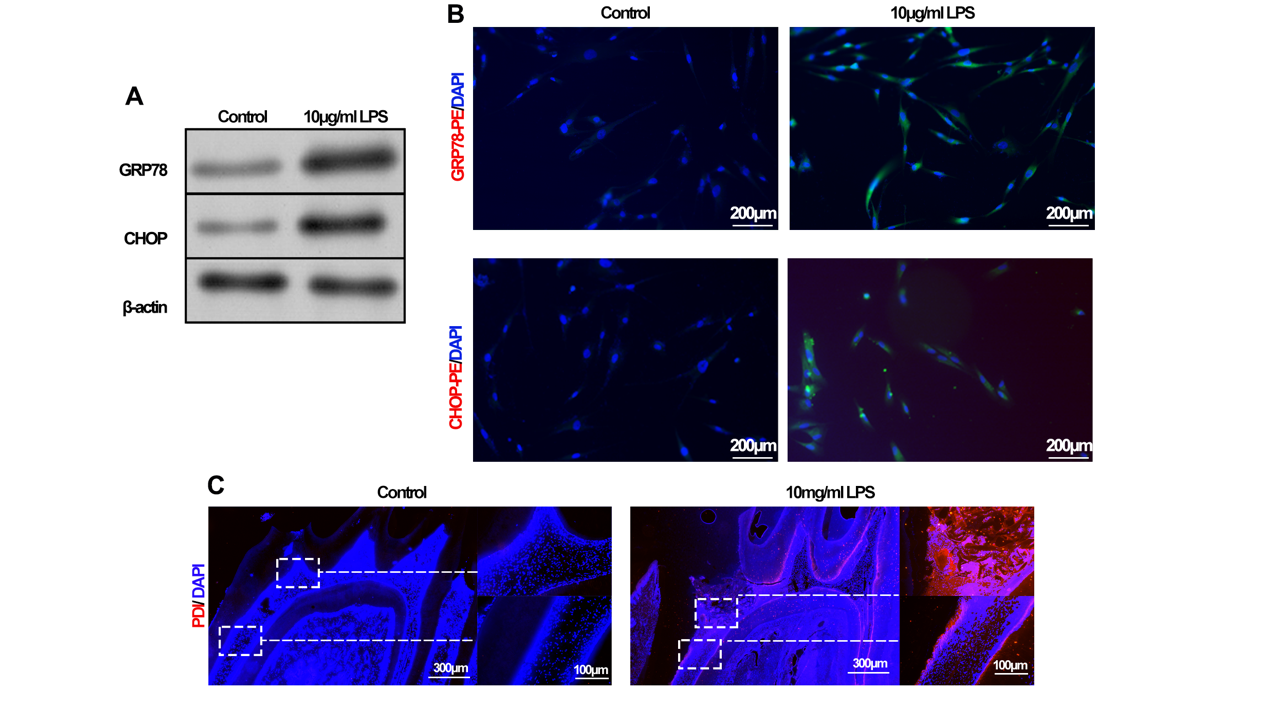


**Figure S6. Pulp injury causes endoplasmic reticulum stress in DPSCs.**

(A) WB results of ERS-related proteins GRP78 and CHOP after LPS treatment. (B) Immunofluorescence results of ERS-associated protein GRP78 and CHOP. (C) PDI immunofluorescence staining of DPSCs at proximal and distal end of injury site, the right panel shows the higher magnification field of view in the white box in the left panel. **P* < 0.05, ***P* < 0.01, ****P* < 0.001, n = 5.


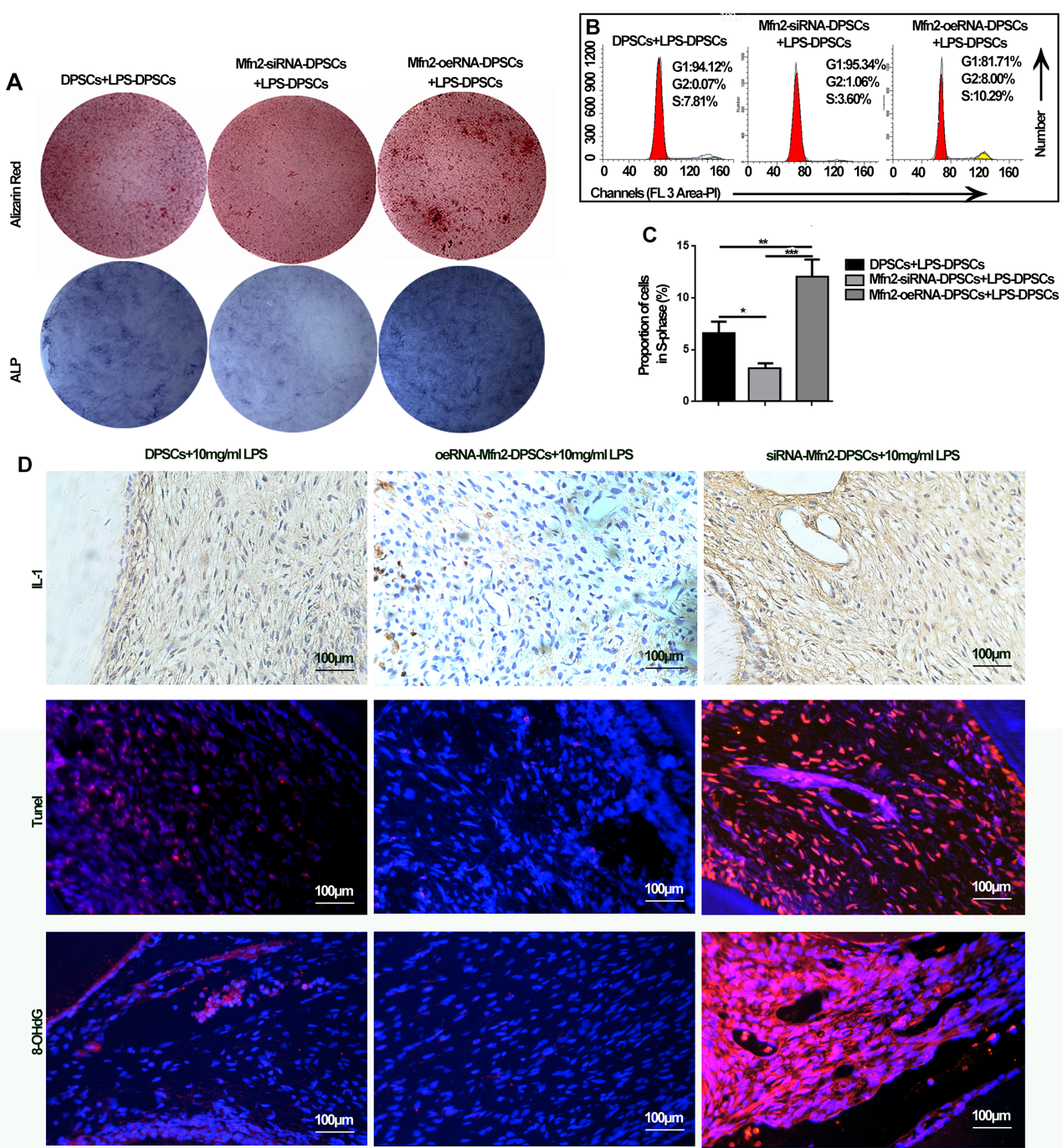


**Figure S7. Mfn2 regulate the effects of mitochondrial transfer on DPSCs function.**

(A) Results of alizarin red and ALP staining of DPSCs after ER regulation of mitochondrial transfer. (B) Flow cytometric cycle results of DPSCs after ER regulation of mitochondrial transfer. (C) Statistical analysis of the flow cytometric cycle of DPSC after ER and lysosomal regulation of mitochondrial transfer. (D)Results of IL-1β immunohistochemistry, TUNEL apoptosis staining and 8-OHdg after mitochondrial transfer. n = 5. **P* < 0.05, ***P* < 0.01, ****P* < 0.001, n = 5.


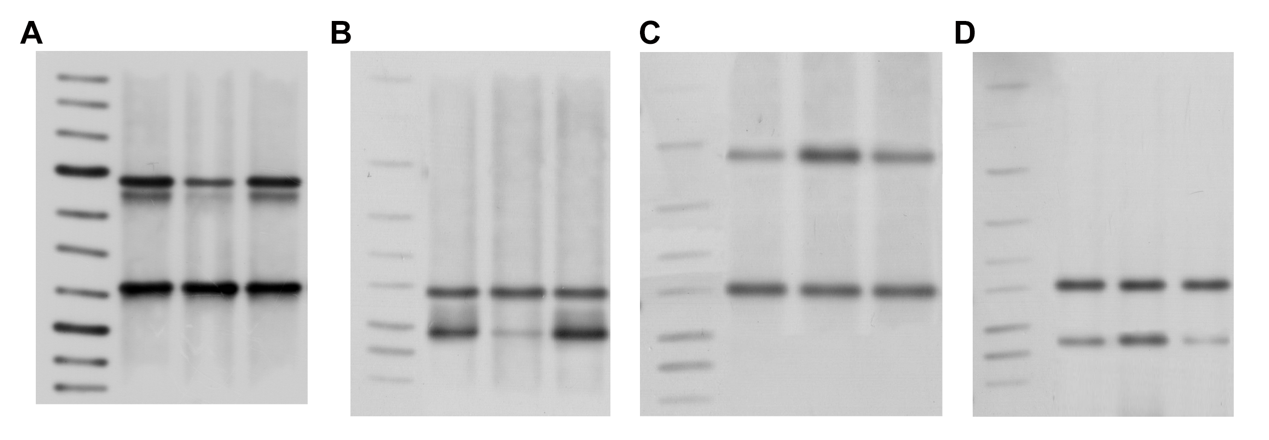


**Figure S8. Full-length gels of western blot in Figure 4.** (A) GRP78. (B) CHOP. (C) GRP78. (D) CHOP

**Movie S1 (.mp4 format). Healthy DPSCs mitochondria (mito-mRFP, Red) dynamically move to 5 μg/ml LPS-DPSCs (CFSE-GFP, Green)**

**Movie S2 (.mp4 format). Healthy DPSCs mitochondria (mito-mRFP, Red) dynamically move to 10 μg/ml LPS-DPSCs (CFSE-GFP, Green)**

**Movie S3 (.mp4 format). Dynamic movement of healthy DPSCs mitochondria (mito-mRFP, Red) and endoplasmic reticulum (ER-RFP, Blue) to LPS-DPSCs (CFSE-GFP, Green)**

**Movie S4 (.mp4 format). Mitochondrial motility of DPSCs**

**Movie S5 (.mp4 format). Mitochondrial motility of Mfn2-siRNA-DPSCs**

**Movie S6 (.mp4 format). Mitochondrial motility of Mfn2-oeRNA-DPSCs**

**Movie S7 (.mp4 format). Mitochondrial motility of mitochondrial transfer donor DPSCs（5μg/ml LPS）**

**Movie S8 (.mp4 format). Mitochondrial motility of mitochondrial transfer donor DPSCs（10μg/ml LPS）**
